# Rapid and affordable size-selected PacBio single-molecule real-time sequencing template library construction using the bead-beating DNA extraction method

**DOI:** 10.14440/jbm.2017.169

**Published:** 2017-09-13

**Authors:** Kengo Kato, Masanori Hashino, Tamaki Ito, Mari Matsui, Satowa Suzuki, Kumiko Kai, Miwako Kitazume, Tsuyoshi Sekizuka, Makoto Kuroda

**Affiliations:** 1 Laboratory of Bacterial Genomics, Pathogen Genomics Center, National Institute of Infectious Diseases, 1-23-1 Shinjyuku-ku, Tokyo 162-8640, Japan; 2 Department of Bacteriology II, National Institute of Infectious Diseases, Musashimurayama, Tokyo 208-0011, Japan; 3 Tomy Digital Biology Co., ltd., 3-14-17 Tagara, Nerima-ku, Tokyo 179-0073, Japan

**Keywords:** bead-beating, library preparation, PacBio, SMRT sequencing

## Abstract

This study demonstrated that bead-beating method facilitates a simple and rapid protocol for genomic DNA isolation for Pacific BioSciences (PacBio) sequencing with library construction of sufficient length. The protocol may also be beneficial for inactivating pathogens by simultaneous and instant DNA fragmentation, with no special equipment required to obtain large DNA fragments. This protocol was comparable in terms of quality to the standard protocol suggested by PacBio and represents an alternative, rapid shortcut for performing accurate PacBio sequencing.

## INTRODUCTION

Next-generation sequencing has expanded the approaches available in the field of genomics [1,2] and facilitates rapid data acquisition of whole genome sequences for living organisms as well as for fossils and mummies [3,4]. Generally, second-generation sequencing (SGS) technology provides high-throughput sequence redundancy for whole genome or metagenomic experiments; however, limitations of this short-read sequencing technology include demonstrating target-specific structural variations or achieving complete genome sequences. Unlike SGS, single-molecule real-time (SMRT) sequencing developed by Pacific BioSciences (PacBio) provides long-read and contiguous sequences, and current fourth-generation chemistry (P6 polymerase and C4 chemistry) methods generate average read lengths of > 10 kb, with an N50 of > 20 kb and a maximum read length of > 60 kb [5].

Genomic DNA isolation, with low damage and sufficient length, prior to SMRTbell library preparation is required to obtain appropriate sequencing results from PacBio sequencing [5]. DNA isolation and subsequent fragmentation steps are time-consuming and expensive because they require an isolation kit for DNA purification and fragmentation using equipment, such as a g-TUBE or a Megaruptor shearing machine (currently unsupported protocols). Here, we demonstrated a simple protocol for size-selected DNA purification using bead-beating and agarose gel electrophoresis, which facilitates SMRTbell library preparation for PacBio sequencing and can reduce the cost and time for processing.

## METHODS

**Figure 1.**
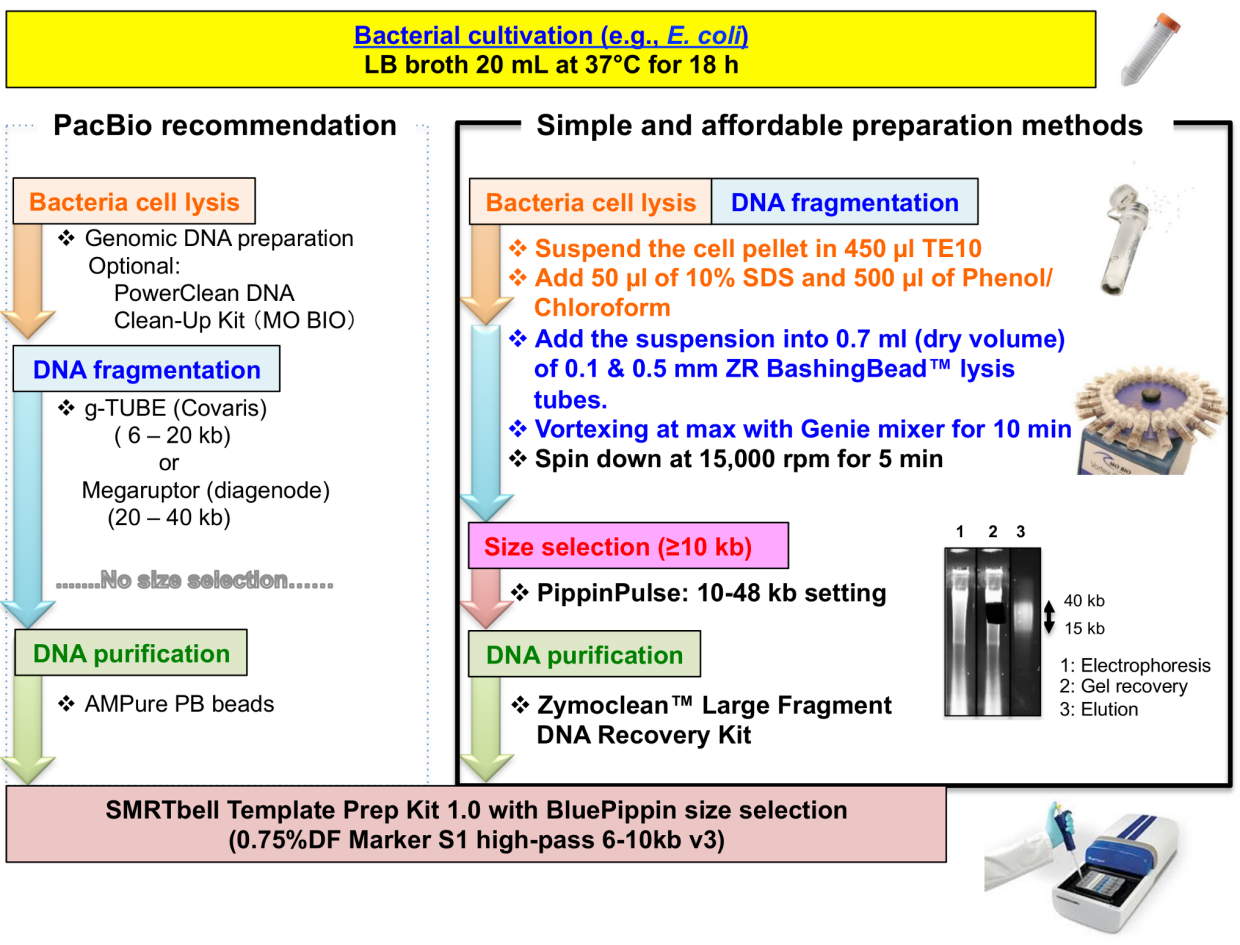
Simple and affordable genomic DNA extraction for SMRTbell library preparation. The standard protocol recommended by PacBio is shown in the left panel. The rapid protocol for cell lysis, DNA fragmentation, and size selection to obtain size-selected DNA fragments for SMRTbell library preparation is shown in the right panel.

Genomic DNA was isolated as shown in **Figure 1.** Bacterial cells were collected from a 20 ml overnight culture, and the cell pellet was suspended with 450 µl TE10 [10 mM Tris (pH 8.0) and 10 mM EDTA]. The cell suspension was supplemented with 50 µl SDS and 500 µl phenol/chloroform, followed by bead-beating for 10 min by vortexing in ZR BasingBead lysis tubes (Zymoresearch, Irvine, CA, USA) attached in a vortex adapter (MO BIO Laboratories, QIAGEN, Carlsbad, CA, USA). After centrifugation at 15000 rpm for 5 min, the upper phase obtained was subjected to electrophoresis on a 1% TAE agarose gel using a Pippin Pulse power supply (Sage Science, Beverly, MA, USA) with a preset running protocol for 10–48 kb (80 V for 9 h using 0.5 × KBB running buffer). The agarose gel was stained with a fluorescent DNA-intercalating dye (*e.g.*, GelRed), and bands within a targeted DNA size (15–40 kb) were excised under a blue light transilluminator (SafeImager; Thermo Fisher Scientific, Waltham, MA, USA) to avoid DNA damage. DNA was purified using a Zymoclean large-fragment DNA recovery kit (Zymoresearch). Purified DNA (~2.0 µg) was used to prepare a SMRTbell library using the SMRTbell template prep kit 1.0 (PacBio, Menlo Park, CA, USA) according to manufacturer instructions.

The SMRTbell library was evaluated using a fragment analyzer (Advanced Analytical, Ankeny, IA USA) to determine the DNA length of the library. SMRT sequencing was performed for one library on one SMRT cell v3 using P6C4 chemistry on a PacBio RSII sequencer. The obtained raw polymerase reads were analyzed using HGAP v3.0 pipeline based on Celera *de novo* assembler and Quiver polishing scripts [6].

S1-pulsed-field gel electrophoresis (PFGE) was performed as described previously [7], followed by plasmid DNA separation by PFGE. Prior to PFGE, an agarose plug containing bacterial genomic DNA was treated with S1 nuclease to digest circular forms of the plasmids, resulting in their linearized forms.

## RESULTS AND DISCUSSION

Simple experimental procedures are favored for whole-genome sequencing. Particularly, PacBio SMRTbell library preparation is relatively laborious because long DNA with minimal damage and no nicks is required. Here, we demonstrated a very short, simple, and affordable isolation method for genomic DNA that is suitable for PacBio sequencing. Bead-beating simultaneously and rapidly produces bacterial lysis and genomic DNA fragmentation, with no special equipment required to obtain large DNA fragments (**Fig. 1**). In this study, we demonstrated library preparation for *E. coli*. Although we performed agarose gel electrophoresis using a Pippin Pulse unit, conventional 1% TAE-agarose gel electrophoresis could alternatively be used to recover large genomic DNA fragments.

**Figure 2.**
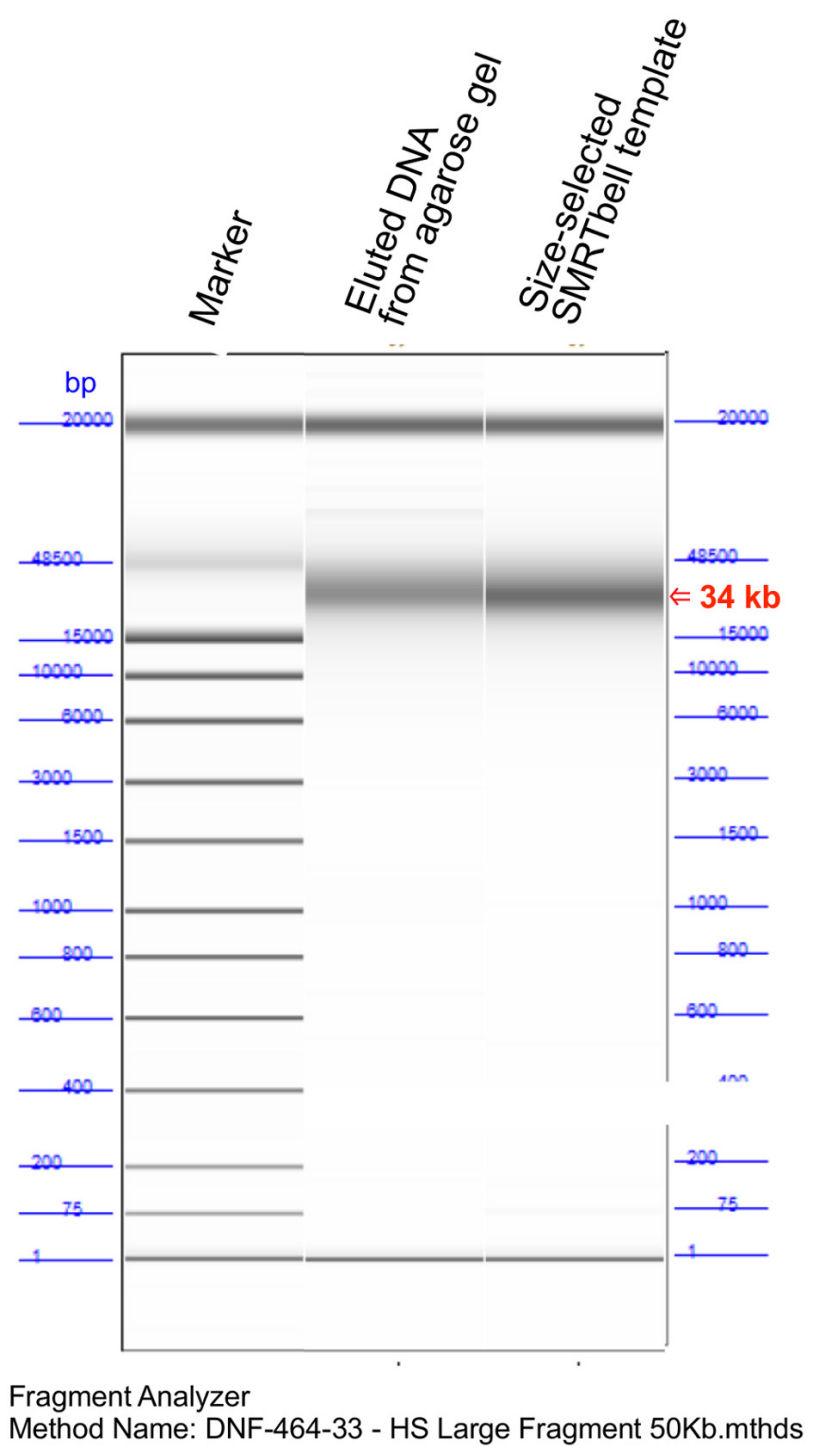
Fragment analysis of the SMRTbell library. Quality of the purified DNA and the subsequent SMRTbell library was evaluated by the Fragment Analyzer.

**Figure 3.**
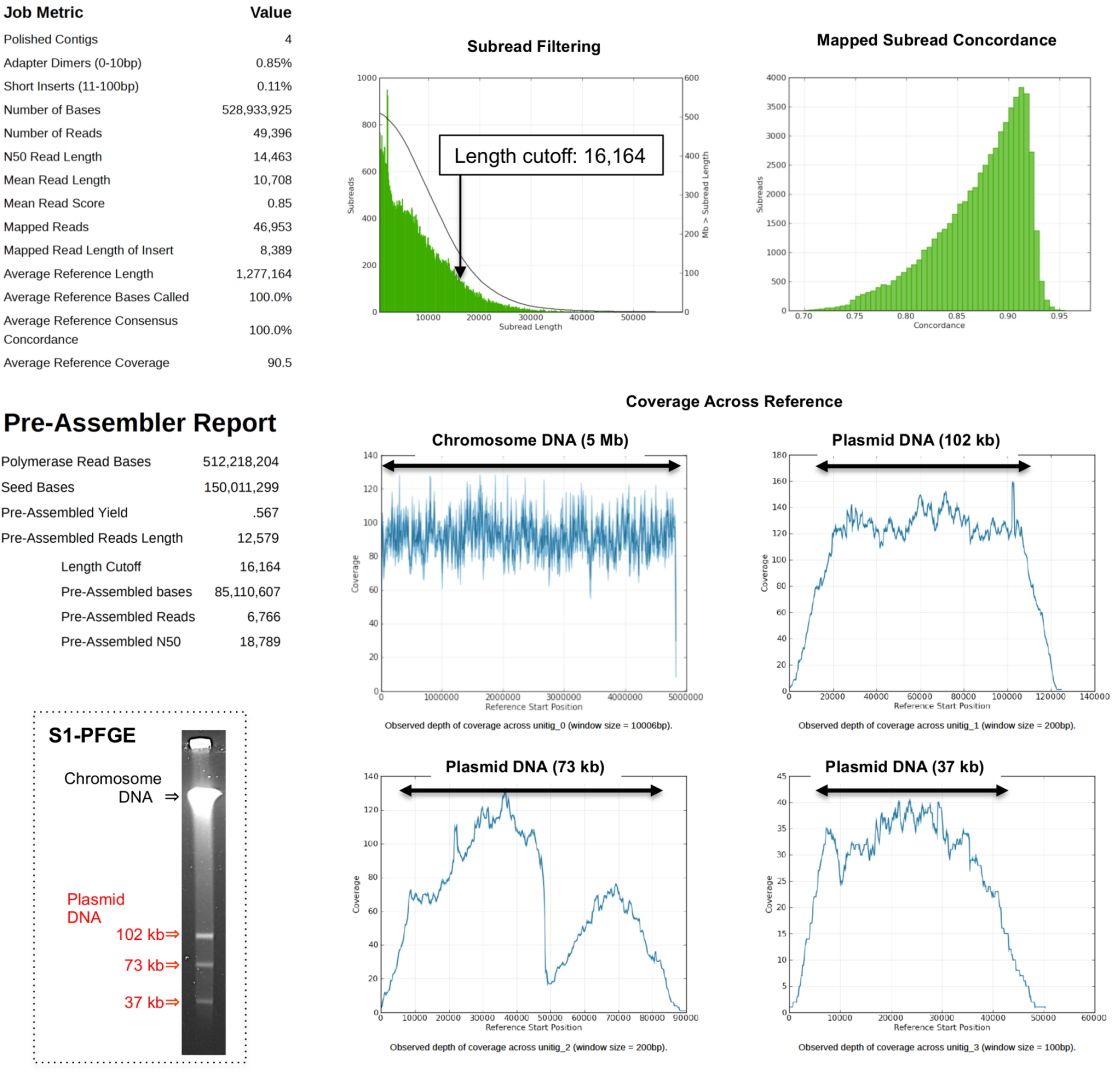
The status of raw sequencing reads and HGAP assemblies. SMRT sequencing of the library (**Fig. 2**) was performed on a single SMRT cell, followed by *de novo* assembly using HGAP version 3.0. Seed reads were obtained above a 16164-nt cutoff. Error correction of the seed reads by residual sub-reads and following *de novo* assembly was performed by HGAP version 3.0 resulting in four polished contigs including a single chromosomal DNA and three individual plasmids. Lengths of the three plasmids were consistent with S1-PFGE results.

Purified, size-selected DNA is suitable for SMRTbell library preparation (**Fig. 2**); indeed, the library was well prepared as approximately > 10 kb in length for PacBio sequencing. Raw reads were analyzed using HGAP version 3.0 *de novo* assembler. The “Length Cutoff” value in the pre-assembler report (**Fig. 3**) indicated that 16164 nt was the minimum length of seed reads for HGAP *de novo* assembly. This result suggested that sufficiently long DNA sequence reads could be extracted at the first step of *de novo* assembly, with four polished contigs obtained (**Fig. 3**). The tested *E. coli* isolate harbored three distinct plasmids of 102 kb, 73 kb, and 37 kb (**Fig. 3**), with complete scaffolds obtained as a single contig for the chromosomal DNA (5 Mb, ~90 × depth) and three contigs for the respective plasmids. Contig lengths were consistent with the size predicted by S1-PFGE (**Fig. 3**) suggesting that the sequencing strategy used in this study achieved complete scaffolding for extrachromosomal DNA, such as plasmids.

Recently, a similar rapid technique was reported by Mayionade *et* *al*. [8] although their method did not state sufficient homogenization conditions using liquid nitrogen and was not applicable for pathogens or clinical specimens due to the risk of infectious diseases. By contrast, this study produced whole-genome sequencing of a pathogenic *E. coli* strain accompanied by inactivation of potential pathogens with phenol/chloroform extraction. Addition of phenol results in complete inactivation of most bacterial pathogens and allows protein removal [9] offering dual benefits for simultaneous DNA fragmentation and pathogen inactivation. Additionally, Gram-positive bacteria, such as *Staphylococcus* spp., can be processed to obtain sufficient amounts of genomic DNA without pretreatment by a bacterial lysis reaction using lysozyme or achromopeptidase (data not shown). We have performed this method successfully for > 50 times for Gram-negative and -positive bacterial-genome scaffolding using the PacBio RSII and Sequel platforms. Furthermore, this rapid protocol can theoretically be applied to any living cells/tissues and metagenomic samples (*e.g.*, soil and/or sewage sediment) for large DNA isolations.

PacBio recommends DNA fragmentation using a g-TUBE or Megaruptor shearing machine as the initial step, but no size selection is indicated prior to following the SMRTbell library preparation protocol (**Fig. 1**). We have occasionally isolated highly fragmented genomic DNA with conventional genomic DNA purification because of over-growth associated with bacterial cultivation or the activity of endogenous nucleases implying that the sheared DNA solution includes short DNA fragments that may reduce the efficiency of PacBio adapter ligation due to short-fragment contamination. This could relatively limit ligation performance; therefore, genomic DNA has to be purified to the range of target sizes in advance. Overall, the raw sequence reads obtained in this study were of comparable quality to those obtained using the standard protocol suggested by PacBio; therefore, our rapid protocol represents a rapid alternative shortcut for accurate PacBio sequencing.
